# Identification and Validation of Candidate Gene Module Along With Immune Cells Infiltration Patterns in Atherosclerosis Progression to Plaque Rupture *via* Transcriptome Analysis

**DOI:** 10.3389/fcvm.2022.894879

**Published:** 2022-06-22

**Authors:** Jing Xu, Cheng Chen, Yuejin Yang

**Affiliations:** ^1^State Key Laboratory of Cardiovascular Diseases, Fuwai Hospital and National Center for Cardiovascular Diseases, Beijing, China; ^2^Chinese Academy of Medical Sciences, Peking Union Medical College, Beijing, China

**Keywords:** atherosclerosis, plaque rupture, microarray, machine learning, immune cells

## Abstract

**Objective:**

To explore the differentially expressed genes (DEGs) along with infiltrating immune cells landscape and their potential mechanisms in the progression of atherosclerosis from onset to plaque rupture.

**Methods:**

In this study, three atherosclerosis-related microarray datasets were downloaded from the NCBI-GEO database. The gene set enrichment analysis (GSEA) was performed for interpreting the biological insights of gene expression data. The CIBERSORTx algorithm was applied to infer the relative proportions of infiltrating immune cells of the atherosclerotic samples. DEGs of the datasets were screened using R. The protein interaction network was constructed *via* STRING. The cluster genes were analyzed by the Cytoscape software. Gene ontology (GO) enrichment was performed via geneontology.org. The least absolute shrinkage and selection operator (LASSO) logistic regression algorithm and receiver operating characteristics (ROC) analyses were performed to build machine learning models for differentiating atherosclerosis status. The Pearson correlation analysis was carried out to illustrate the relationship between cluster genes and immune cells. The expression levels of the cluster genes were validated in two external cohorts. Transcriptional factors and drug-gene interaction analysis were performed to investigate the promising targets for atherosclerosis intervention.

**Results:**

Pathways related to immunoinflammatory responses were identified according to GSEA analysis, and the detailed fractions infiltrating immune cells were compared between the early and advanced atherosclerosis. Additionally, we identified 170 DEGs in atherosclerosis progression (|log2FC|≥1 and adjusted *p* < 0.05). They were mainly enriched in GO terms relating to inflammatory response and innate immune response. A cluster of nine genes, such as *ITGB2, C1QC, LY86, CTSS, C1QA, CSF1R, LAPTM5, VSIG4*, and *CD163*, were found to be significant, and their correlations with infiltrating immune cells were calculated. The cluster genes were also validated to be upregulated in two external cohorts. Moreover, *C1QA* and *ITGB2* may exert pathogenic functions in the entire process of atherogenesis.

**Conclusions:**

We reanalyzed the transcriptomic signature of atherosclerosis development from onset to plaque rupture along with the landscape of the immune cell, as well as revealed new insights and specific prospective DEGs for the investigation of disease-associated dynamic molecular processes and their regulations with immune cells.

## Introduction

Atherosclerosis, which refers to the subendothelial accumulation of lipids that trigger maladaptive immune responses, is the major cause of global cardiovascular disease, ischemic stroke, and peripheral arterial disease (1, 2). In the past 30 years, a convincing body of experimental and clinical data has illustrated the molecular mechanisms underlying the pathogenesis of atherosclerosis and suggested the most effective for the prevention and treatment of atherosclerosis such as statins and vaccine-based strategies (3). However, despite this progress, the inflammatory nature of atherosclerosis remains a predominant reason for plaque vulnerability (2), and the interplay between immune cells and genetic modulations in atherosclerosis formation, progression, and plaque rupture is still vastly under-investigated (3, 4).

The rapid and enthusiastic adoption of transcriptome analysis technologies has extensively facilitated the detection of biological variation in gene expression, which might be an important molecular phenotype that can affect the physiological parameters (5). Here, we conducted a series of gene expression profiling analyses and performed a machine learning algorithm to investigate the remarkable gene signatures associated with alterations of infiltrating immune cells in the progression of atherogenesis and validated the results in two external datasets representing atherosclerosis from onset to rupture, which may contribute to a better understanding of the pathophysiological mechanisms and the development of curative therapies.

## Materials and Methods

### Data Collection and Preprocessing

**Figure 1:**
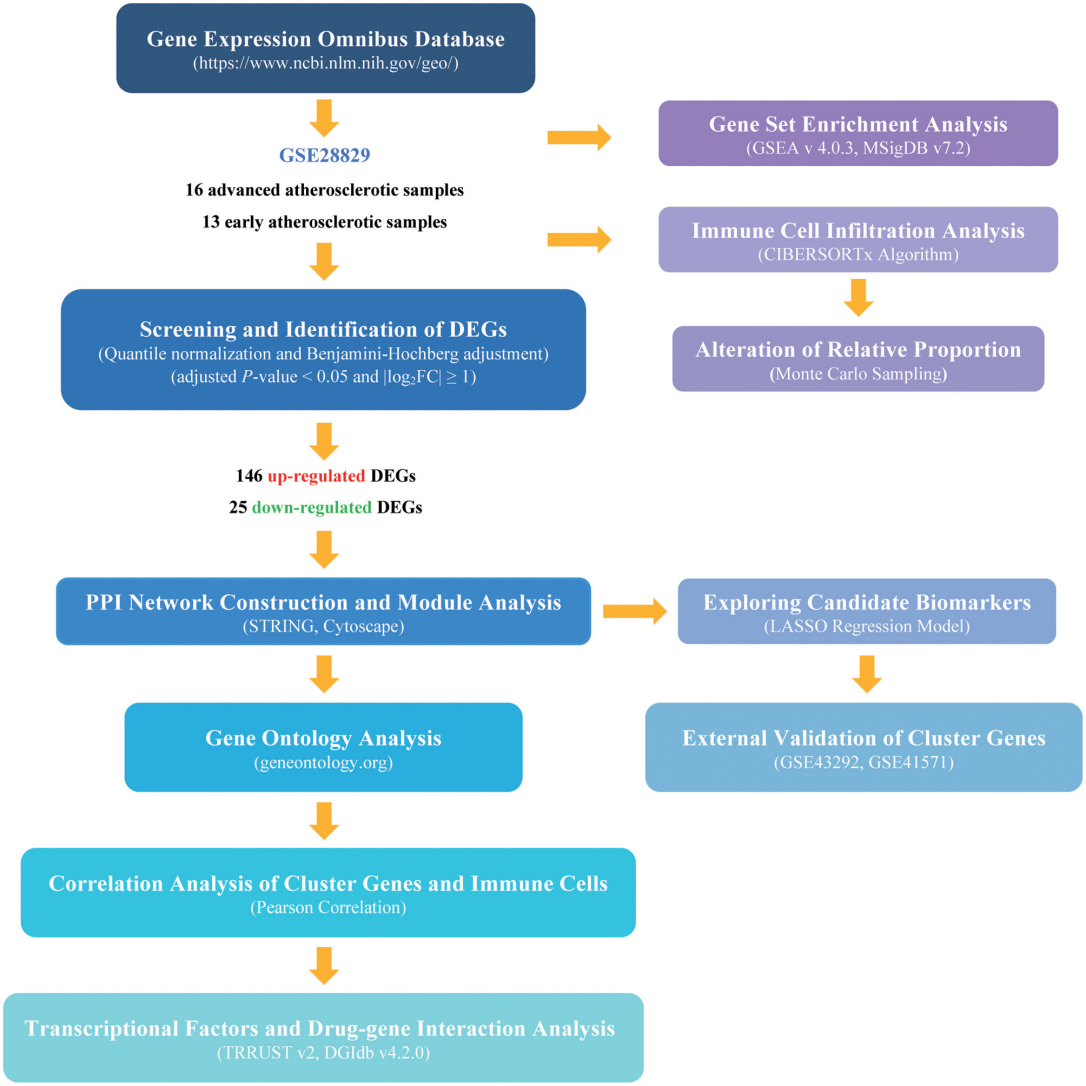
The flowchart of the analysis procedure. The detailed information was described in the materials and methods section.

The processed data files of microarray were downloaded from the Gene Expression Omnibus (GEO) database (https://www.ncbi.nlm.nih.gov/geo/), a global publicly available repository for genomic data submitted by the scientific community (6). The GSE28829 dataset, tested on GPL570 based on Affymetrix Human GenomeU133 Plus 2.0 Array, is the gene expression data of complete transcriptomics analysis of atherosclerotic carotid artery segments. In total, 13 early lesions (pathological intimal thickening and intimal xanthoma), together with 16 advanced lesions (thin or thick fibrous cap atheroma) are enrolled in this study (7). Gene probes were matched to the official gene symbols after the platform reference matrix files were downloaded. For the situation that more than one probe was matched to a gene symbol, we retained the average gene expression value. The following procedures were analyzed according to the processed matrix file. The flowchart of our analysis was shown in Figure 1.

### Gene set Enrichment Analysis and Evaluation of Immune Cells Infiltration

The normalized gene expression data with the matched gene symbols were utilized to perform Gene Set Enrichment Analysis (GSEA) for interpreting the biological insights of gene expression data (8). We downloaded the GSEA software (http://software.broadinstitute.org/gsea/index.jsp, version 4.0.3) and the reference gene sets C2 (c2.cp.kegg.v7.4.symbols.gmt) from the Molecular Signatures Database (MSigDB, http://www.gsea-msigdb.org/gsea/downloads.jsp) to perform GSEA analysis. The normalized enrichment scores (NES) and nominal *p* (NP) values were generated automatically by GSEA. |NES|≥1, *p*-value <0.05, and FDR <0.25 were considered statistically significant. The CIBERSORTx algorithm was applied to infer the relative proportions of infiltrating immune cells of the atherosclerotic samples (9). The matched expression file of GSE28829 was uploaded to the CIBERSORTx online web (http://cibersortx.stanford.edu), with the algorithm run by setting the default signature matrix at 1,000 permutations for quantifying immune cell fractions from bulk tissue gene expression profiles. The CIBERSORTx generates a *p*-value for the deconvolution for each sample in the results using the Monte Carlo sampling. The significant alteration of immune cells between early and advanced atherosclerosis was identified according to the threshold of the *Wilcoxon test* at *p* < 0.05.

### Identification of the Differentially Expressed Genes (DEGs)

The *limma* package was used to screen DEGs between early and advanced atherosclerosis based on the R platform (R-project.org, version 3.6.1) (10). Fold change (FC) by logarithmic operations with 2 as base numbers were used to make easier calculations and more scientific comparisons for the expression of each gene between early and advanced atherosclerotic samples. Genes with |log_2_FC|≥1 were considered as DEGs, and statistical significance was defined by adjusted *p*-value < 0.05, corrected by the *Benjamini-Hochberg* method.

### Protein Interaction and Module Analysis

Search Tool for the Retrieval of Interacting Genes (STRING, http://string-db.org/, version 11.5) database was used to analyze the protein-protein interaction (PPI) of the DEGs (11). The DEGs were entered into the identifier and *Homo sapiens* were selected as the organism. To further narrow the candidate gene field, a high confidence level of 0.700 was assessed. Next, the PPI networks were constructed and visualized using the Cytoscape software (version 3.8.2) (12). The plug-in Molecular Complex Detection (MCODE, version 2.0.0), an automated kit to find densely connected regions based on topology, was used to screen the co-expression network of atherosclerosis(13). The MCODE parameters criteria were set by default as follows: degree cut-off = 2, node score cut-off = 0.2, Max depth = 100, and k-score = 2.

### Functional Enrichment Analysis of the Cluster Genes

The gene ontology (GO) analysis is an international gene functional classification system for determining the correlation between the selected genes and standardized categories through a hypergeometric test (14). The GO enrichment analysis of the cluster genes such as biological process (BP), cellular component (CC), and molecular function (MF) was performed *via* http://geneontology.org (15). Particularly, *Homo sapiens* were selected in order to limit the annotation of the species. A *p*-value of < 0.05 was considered a threshold value.

### Correlation Analysis of Genes and Immune Cells

Pearson correlation analysis was applied to explore the relationship between gene expressions and the relative proportion of immune cells in early and advanced atherosclerosis samples analyzed by the CIBERSORTx (16). Based on the paired *t*-test, *p* < 0.05 was considered statistically significant.

### Prediction Model Analysis *via* Lasso Cox Regression

Lasso Cox regression analysis was performed *via* the *glmnet* package in R software to calculate and select the linear models and preserve valuable variables (17). The expression level of cluster DEGs and the diagnosis of 29 samples were obtained from the probe-matched matrix file, and the samples were randomly assigned to training or testing cohorts in approximately a 2:1 ratio. Variables with calculated coefficients were used to build the classification model. The *pROC* package in R software was used for the display of Receiver Operating Characteristics (ROC) analysis and the calculation of the area under the curve (AUC) (18). Thus, we investigated the prediction feasibility of hub genes *via* the AUC values.

### External Validation of the Hub Genes

For the validation of the identified hub genes, GSE43292 and GSE41571 datasets were downloaded from the GEO database. GSE43292 contains 32 samples of non-atherosclerotic tissue and 32 samples of atheroma plaque, and GSE41571 contains 5 samples of ruptured plaque and 6 samples of stable plaque (19, 20). The expressions of the cluster genes were extracted from the microarray dataset and analyzed by student's *t*-test. *p*-value < 0.05 was defined as statistical significance. The ROC curve was constructed to identify the discriminatory power of selected biomarkers correlated to atherosclerosis progression and plaque rupture.

### Transcriptional Factors and Drug-Gene Interaction Analysis

The TRRUST v2 database (www.grnpedia.org/trrust) was applied to analyze the key regulators in transcriptional factors (TFs)-gene interactions to assess the effect of the overlapped TFs on the expression and functional pathways of the cluster genes (21). The cluster genes also served as promising targets for searching for candidate drugs through the Drug-Gene Interaction database (DGIdb, http://www.dgidb.org/, v4.2.0—sha1 afd9f30b) (22).

## Results

### GSEA Analysis Based on the Integral Expression Data

**Figure 2:**
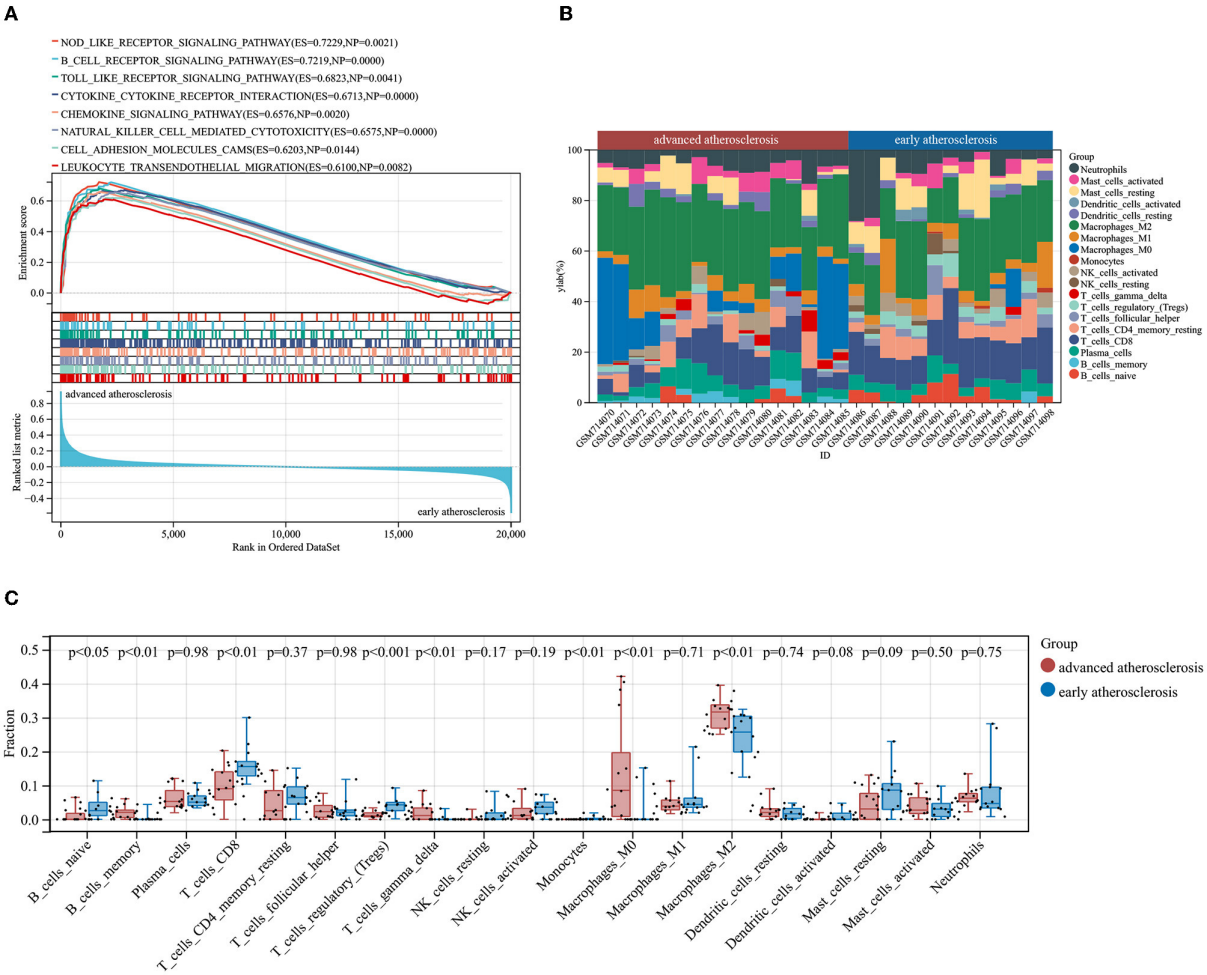
Results of GSEA and CIBERSORTx analysis of GSE28829. **(A)** Enriched KEGG pathways in advanced atherosclerosis. ES, enrichment score; NP, nominal *p*-value. **(B)** The immune cells landscape of the samples in GSE28829. **(C)** The alterations of infiltrating immune cells between early and advanced atherosclerosis.

First, we performed GSEA analysis considering all the gene expressions in GSE28829, not only those above the cutoff in terms of fold change or significance. We noticed that a large number of annotated Kyoto Encyclopedia of Genes and Genomes (KEGG) pathways enriched in advanced atherosclerosis were correlated with immunoinflammatory responses including “NOD_LIKE_RECEPTOR_SIGNALING_PATHWAY” (ES = 0.7229, NP = 0.0021); “B_CELL_RECEPTOR_SIGNALING_PATHWAY” (ES = 0.7219, NP <0.001); “TOLL_LIKE_RECEPTOR_SIGNALING_PATHWAY” (ES = 0.6823, NP = 0.0041); “CYTOKINE_CYTOKINE_RECEPTOR_INTERACTION” (ES = 0.6731, NP <0.001); “CHEMOKINE_SIGNALING_PATHWAY” (ES = 0.6576, NP = 0.0020); and “NATURAL_KILLER_CELL_MEDIATED_CYTOTOXICITY” (ES = 0.6576, NP <0.001). Additionally, other terms including “CELL_ADHESION_MOLECULES_CAMS” (ES = 0.6203, NP = 0.0144), “LEUKOCYTE_TRANSENDOTHELIAL_MIGRATION” (ES = 0.6100, NP = 0.0082), as well as autoimmune-related terms including “SYSTEMIC_LUPUS_ERYTHEMATOSUS”(ES = 0.7604, NP = 0.0061), “AUTOIMMUNE_THYROID_DISEASE” (ES = 0.7269, NP = 0.0021), and “ASTHMA” (ES = 0.7835, NP = 0.0063) were also annotated with significance, indicating the multifaceted mechanisms underlying the progression of atherosclerosis (Figure 2A, see Supplementary Table 1 for details) (3, 4).

### Evaluation of Immune Cell Infiltration

According to the results of GSEA analysis, the immune cells may play a causative role in triggering atherosclerotic plaque progression. Therefore, we performed the CIBERSORTx algorithm to comprehensively characterize the infiltration percentages of 19 detectable subpopulations of immune cells in the early and advanced atherosclerotic samples from GSE28829. The relative percentage of each cell in 13 early lesions and 16 advanced lesions is shown in Figure 2B. Moreover, as shown in Figure 2C, the relative proportions of 8 subtypes of the immune cells were significantly different and objectively detectable between early and advanced atherosclerotic samples. Memory B cells (*p* < 0.01), gamma delta T cells (*p* < 0.01), M0 macrophages (*p* < 0.01), and M2 macrophages (*p* < 0.01) had a higher fraction in advanced atherosclerosis samples, while naïve B cells (*p* < 0.05), CD8+ T cells (*p* < 0.01), regulatory T cells (Tregs, *p* < 0.001), and monocytes (*p* < 0.01) contained a lower fraction in advanced atherosclerosis samples. The detailed results of the CIBERSORTx analysis and measures of confidence are shown in Supplementary Table 2.

### Identification of the DEGs Between Early and Advanced Atherosclerotic Samples

The available numerical expression values of 13 early lesions and 16 advanced lesions from GSE28829 were used to identify the DEGs. Compared with the early atherosclerosis samples, a total of 171 DEGs including 146 up-regulated and 25 down-regulated DEGs were screened in advanced atherosclerosis samples (|log_2_FC| ≥ 1 and adjusted *p*-value < 0.05). The expression data with gene symbols are shown in Supplementary Table 3.

### Protein Interaction and Module Analysis

**Figure 3:**
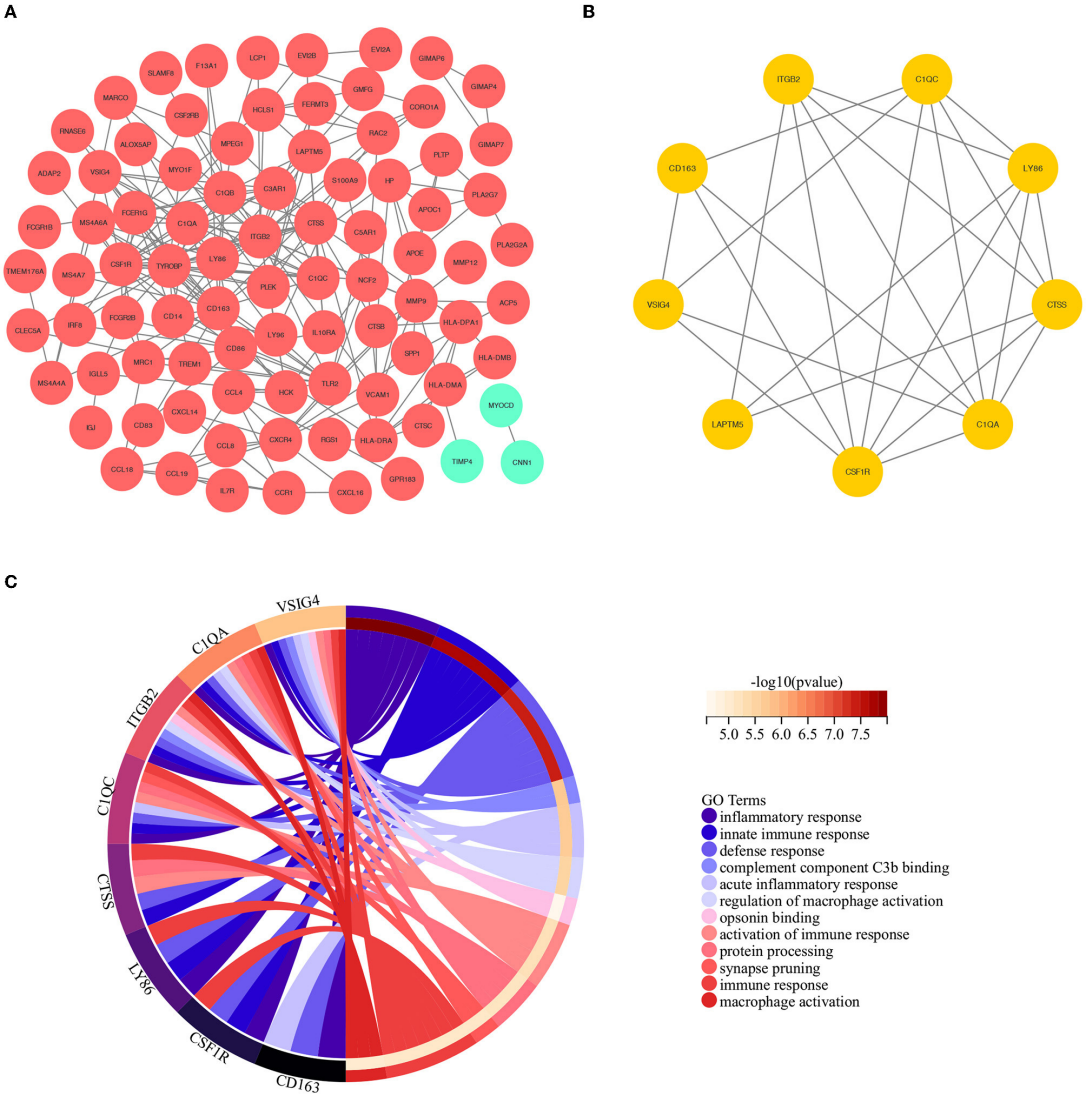
**(A)** The construction of the PPI network based on the DEGs. The red ellipse represents up-regulated DEGs, the green ellipse represents down-regulated DEGs. **(B)** The cluster genes with the highest scores in the PPI network, displayed by the yellow ellipse. **(C)** The results of GO analysis based on the selected cluster genes.

To construct the PPI network of the identified DEGs, the STRING online database and the Cytoscape software were utilized. A total of 171 DEGs were filtered into the PPI network, including 87 nodes and 242 edges (Figure 3A). Based on the high confidence level of 0.70, 84 genes did not fall into the PPI network. According to the node degree> 10 criteria, the 14 hub genes were *TYROBP* (degree = 25), *ITGB2* (degree = 24), *CSF1R* (degree = 21), *CD163* (degree = 17), *C1QB* (degree = 16), *C1QA* (degree = 14), *CTSS* (degree = 13), *FCER1G* (degree = 13), *LY86* (degree = 13),*TLR2* (degree = 13), *CD86* (degree=12), *LAPTM5* (degree = 11), *MMP9* (degree = 11), and *CXCR4* (degree = 10). The plug-in kit MCODE was conducted to analyze the significant module, and a module with 9 nodes and 24 edges was selected from the PPI network (Figure 3B), showing that the cluster genes were composed of *ITGB2, C1QC, LY86, CTSS, C1QA, CSF1R, LAPTM5, VSIG4*, and *CD163*. The descriptions of the cluster genes were listed in Table 1.

**Table 1:** Detailed information of the cluster genes.

**Gene Name**	**Description**	**GSE28829**	
		**Log_**2**_FC**	**Adjusted *p*-value**
*C1QC*	complement C1q C chain	1.7735	0.0003
*LY86*	lymphocyte antigen 86	1.5991	0.0001
*CTSS*	cathepsin S	1.6963	0.0001
*C1QA*	complement C1q A chain	1.4529	7.42E-5
*CSF1R*	colony stimulating factor 1 receptor	1.1724	8.47E-4
*LAPTM5*	lysosomal protein transmembrane 5	1.6249	6.92E-5
*VSIG4*	V-set and immunoglobulin domain containing 4	1.4312	0.0004
*CD163*	CD163 molecule	1.0053	0.0342
*ITGB2*	integrin subunit beta 2	1.4403	3.43E-5

*DEGs were set as |log_2_FC|≥1and adjusted p < 0.05*.

*Description of the gene was obtained from Human Genome Resources at NCBI. The log_2_FC and adjusted p-value were calculated in comparison of advanced atherosclerosis with early atherosclerosis in GSE28829*.

### Functional Annotation and Enrichment of Cluster Genes

To further investigate the biological interpretations of the densely connected genes, GO analysis of the cluster genes was conducted *via* geneontology.org database, and a total of 118 significant GO terms was annotated (adjusted *p*-value of < 0.05, see Supplementary Table 4 for details). Consistent with the results of GSEA analysis, the enriched GO terms were also related to immunoinflammatory responses. The leading GO term based in the highest significance were (1) GO:0006954~inflammatory response (adjusted *p* = 7.09E-6); (2) GO:0045087~innate immune response(adjusted *p* = 8.75E-6); (3) GO:0006952~defense response (adjusted *p* = 1.16E-5); (4) GO:0001851~complement component C3b binding (adjusted *p* = 0.0002); (5) GO:0002526~acute inflammatory response (adjusted *p* = 0.0005); (6) GO:0043030~regulation of macrophage activation (adjusted *p* = 0.0006); (7]) GO:0001846~opsonin binding (adjusted *p* = 0.0008); (8) GO:0002253~activation of immune response (adjusted *p* = 0.0008); (9) GO:0016485~protein processing(adjusted *p* = 0.0008); (10) GO:0098883~synapse pruning (adjusted *p* = 0.0008); (11) GO:0006955~immune response(adjusted *p* = 0.0008); (12) GO:0042116~macrophage activation (adjusted *p* = 0.0009). The relations between cluster genes and the leading GO terms are shown in Figure 3C.

### Correlation Analysis of Genes and Immune Cells

**Figure 4:**
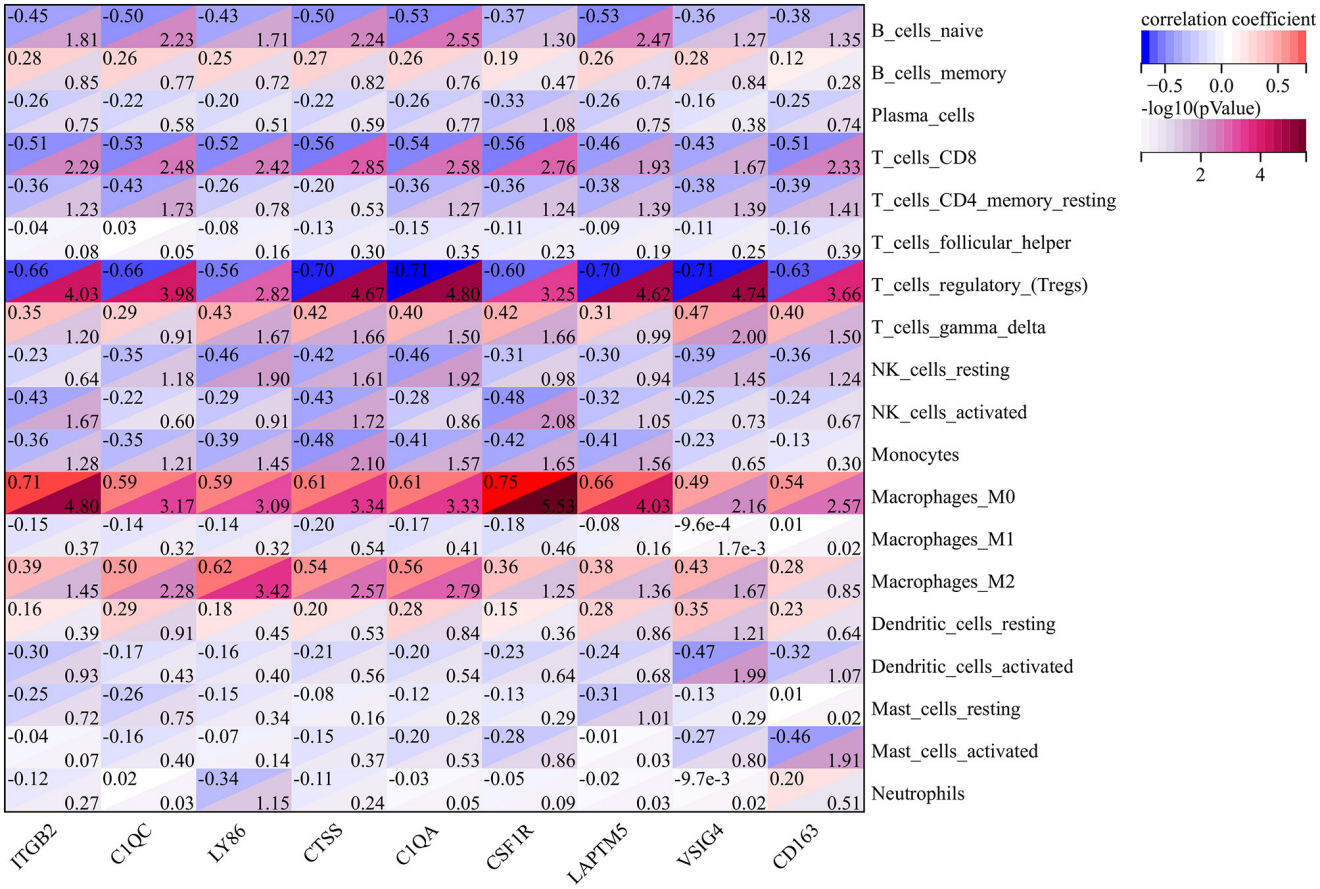
The Pearson Correlation Analysis between the cluster genes and immune cells. The values in the top left corner of the rectangle represent the correlation coefficient, and the values in the lower right corner of the rectangle represent -log10 (*p-*Value).

The results of previous GSEA analysis and GO enrichment annotation both indicated that immune cells may play an important role in the process of atherosclerosis. Therefore, we carried out a Pearson correlation analysis to illustrate the relationship between cluster genes and immune cells in early and advanced atherosclerotic samples. As shown in Figure 4, it is worth noting that at the threshold of R>0.5 and *p* < 0.05, almost all the cluster genes represented negative correlations with Tregs and positive correlations with M0 macrophages. Besides, *ITGB2, C1QC, LY86, CTSS, C1QA, CSF1R*, and *CD163* showed negative correlations with CD8+ T cells, similar correlations were also observed in *C1QC, CTSS, C1QA*, and *LAPTM5* with naïve B cells. Moreover, *C1QC, LY86, CTSS*, and *C1QA* also exerted positive correlations with M2 macrophages. These results further illustrated that cluster genes may promote atherosclerosis via the regulation of immune cell infiltration and differentiation.

### Exploring Candidate Genes by Lasso Cox Regression

**Figure 5:**
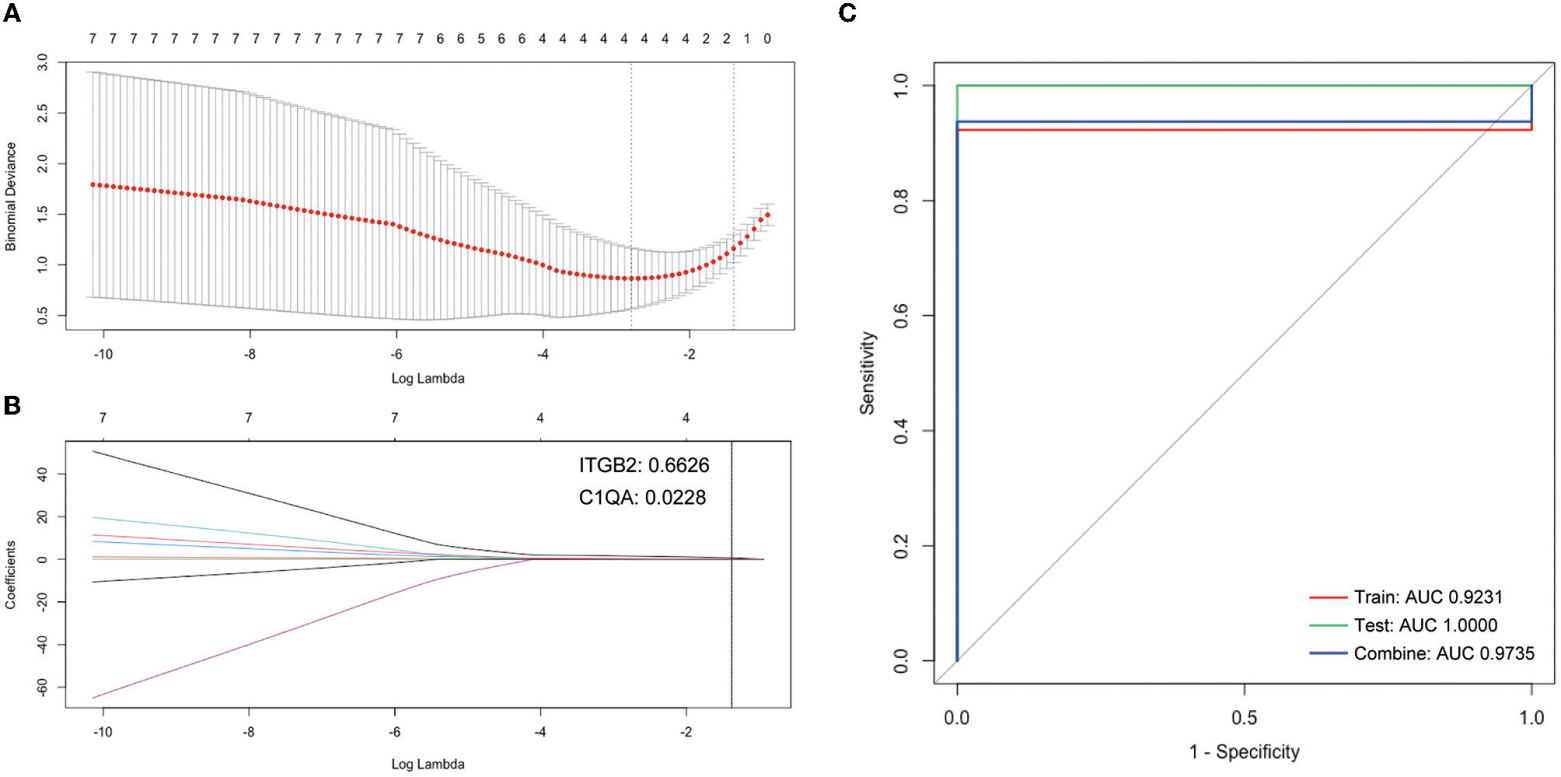
Construction of LASSO cox regression model. **(A)** The red dots represent the values of binomial deviance; the gray lines represent the standard error (SE); the vertical dotted lines represent optimal values by the minimum criteria and 1-SE criteria. “Lambda” represents the tuning parameter. **(B)** The plot determines the coefficient by 1-SE criteria of LASSO regression model 0.0228 and 0.6626 for *C1QA* and *ITGB2*, respectively. **(C)** The ROC curves of the LASSO regression model of training, testing, and combination cohort in GSE28829.

Firstly, the Lasso Cox regression model for the cluster DEGs of atherosclerotic samples from GSE28829 was conducted to investigate an optimum linear combination in predicting advanced atherosclerosis (Figures 5A,B), with coefficients 0.6626 and 0.0228 for *ITGB2* and *C1QA*, respectively. Then, the ROC curve analysis of the Lasso Cox regression classifier was conducted separately to predict advanced atherosclerosis in the training cohort, testing cohort, a combination cohort, and the AUC values all beyond 0.9 (Figure 5C), which suggests it might have a decent potentiality of being biomarkers for screening the progression of the atherosclerosis.

### Cluster Genes Validation Targeting Potential Genes for Atherosclerosis Progression

**Figure 6:**
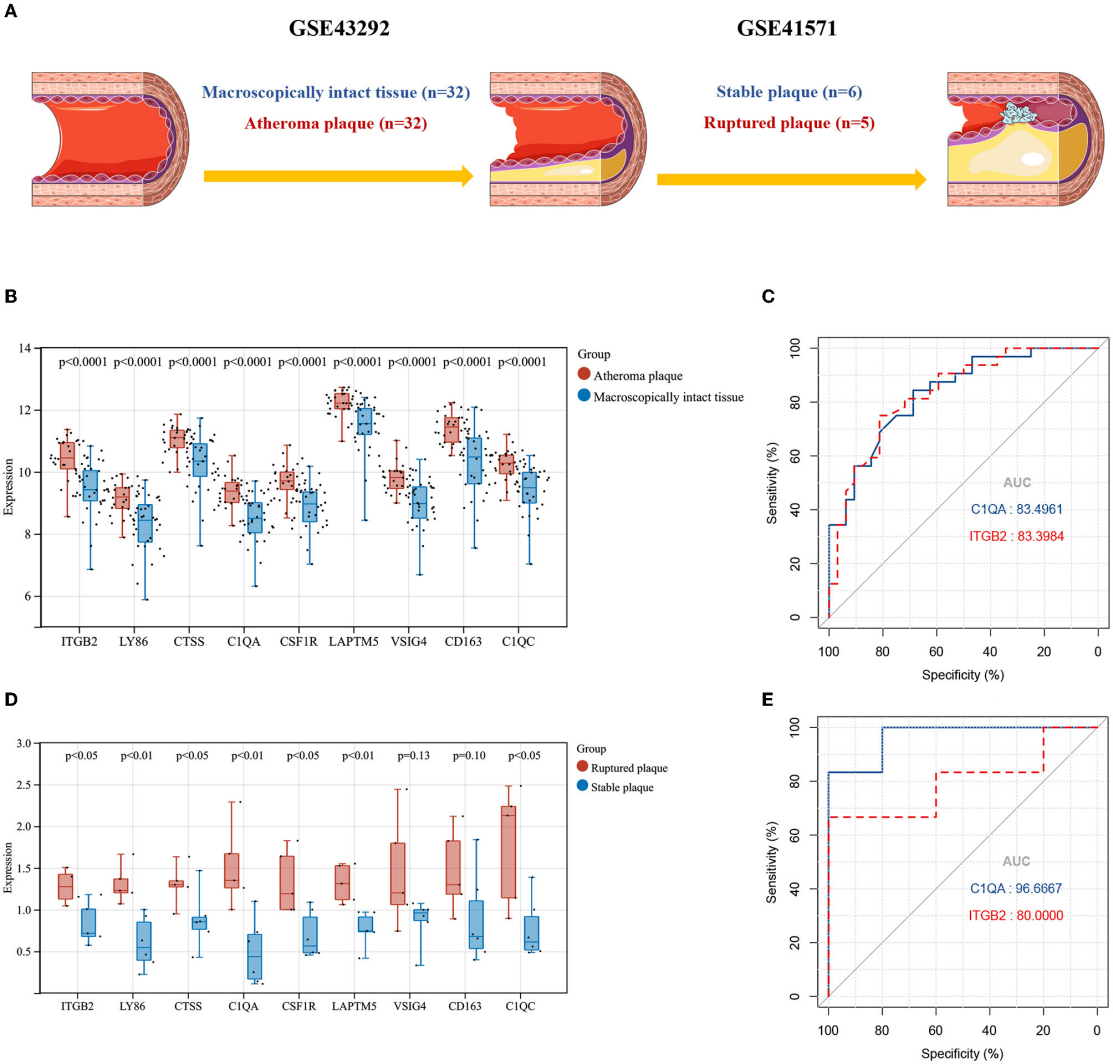
**(A)** The detailed information and the pathophysiological relations of validation datasets GSE43292 and GSE41571. **(B)** The expression levels of the cluster genes in GSE43292and analyzed by student's *t*-test. **(C)** ROC curves for *C1QA* and *ITGB2* in GSE43292. **(D)** The expression levels of the cluster genes in GSE41571 and analyzed by student's *t* test. **(E)** ROC curves for *C1QA* and *ITGB2* in GSE41571. AUC, area under the curve; ROC, receiver operating characteristic.

To further validate the accuracy and reliability of the cluster genes selected from GSE28829, two external cohorts from the GSE43292 and GSE41571 datasets containing were included in our study. The detailed information and pathological relationships between the two datasets were shown in Figure 6A, and the expression values of the cluster genes were extracted and analyzed independently. Consistent with the results of GSE28829, these cluster genes (*n* = 9) were all significantly upregulated in atheroma plaque samples (Figure 6B). We further conducted ROC analysis to investigate the feasibility of *C1QA* and *ITGB2* as prognostic DEGs, and the results showed good predictive accuracy (Figure 6C). For *C1QA*, the AUC was 83.4961% at the optimal cut-off value of 9.115, and the sensitivity and specificity were 84.375% and 68.75%, respectively; for *ITGB2*, the AUC was 83.3984% at the optimal cut-off value of 10.03, with the sensitivity being 75% and specificity being 81.25%.

Similarly, in GSE41571, nine (*n* = 9) of these cluster genes in ruptured plaques were all observed upregulated with seven (*n* = 7) of them being significant except for *VSIG4* and *CD163*, as compared with stable plaques (Figure 6D). For *C1QA*, the sensitivity was 100% and specificity was 83.3333% at the optimal cut-off value of 0.8650, with the AUC being 90.6667%; for *ITGB2*, at the optimal cut-off value of 1.0257, the sensitivity and specificity were 100% and 66.6667%, respectively, and the AUC was 80% (Figure 6E).

Together, the expression levels of cluster genes were validated in two external cohorts, which further demonstrated their importance in the development of atherosclerosis. Additionally, *C1QA* and *ITGB2* could not only differentiate early and advanced atherosclerosis but also highly specific and sensitive DEGs for predicting plaque rupture.

### Transcriptional Factors and Drug-Gene Interaction Analysis

We then attempted to investigate the key regulators of the nine cluster genes, which may be promising targets for atherosclerosis intervention. Three (*n* = 3) key TFs were identified *via* the TRRUST v2 database to potentially regulate the expression of *CD163, CSF1R, CTSS*, and *ITGB2* (Table 2). The drug-gene interaction network of the cluster genes was screened aiming to identify druggable targets. As shown in Table 3, a total of 6 intersecting drugs targeting 3 genes, *CSF1R, CTSS*, and *ITGB2*, were selected as candidate druggable molecular targets for atherosclerosis.

**Table 2:** Key transcriptional factors of the cluster genes.

**Key TF**	**Description**	**List of overlapped genes**	***p*-value**
SPI1	Spleen focus forming virus (SFFV) proviral integration oncogene spi1	*ITGB2, CTSS, CD163*	2.80E-06
RUNX1	Runt-related transcription factor 1	*ITGB2, CSF1R*	0.000156
SP1	Sp1 transcription factor	*ITGB2*, CD163	0.02

*The results were obtained from TRRUST v2. TF, transcriptional factor*.

**Table 3:** Potential druggable molecular targets for atherosclerosis.

**Gene**	**Drug**	**Interaction types & directionality**	**Mechanism of interaction**
ITGB2	ERLIZUMAB	Negative modulator (inhibitory)	Integrin beta-2 negative modulator
ITGB2	MLNM-2201	Inhibitor (inhibitory)	Integrin beta-2 inhibitor
CTSS	PETESICATIB	Inhibitor (inhibitory)	Inhibition
CTSS	ODANACATIB	Inhibitor (inhibitory)	Inhibition
CSF1R	ARRY-382	Inhibitor (inhibitory)	Macrophage colony stimulating factor receptor inhibitor
CSF1R	CABIRALIZUMAB	Inhibitor (inhibitory)	Direct binding

*The results were obtained from DGIdb, the top 2 drugs (based on interaction score) with interaction information were shown in this table*.

## Discussion

Transcriptome analysis enables an expanded knowledge of complex multicellular biological systems, which has become a cornerstone of many research initiatives (23, 24). In this study, we performed a series of bioinformatics analyses to identify the cluster gene module and immune cells infiltration patterns along with the underlying mechanistic pathways in the progression of atherosclerotic plaques. Compared to some published pipelines, we did not simply merge the datasets tested on different microarray platforms, laboratory conditions, and operation personnel. Although some existing algorithms can adjust batch effects *via* modifying the data matrices, it is still insufficient to capture all the batch effects entirely which can cause substantial artifacts and confound the accuracy of analysis, leading to spurious results (25, 26). To this end, we conducted the principal analysis on an independent dataset GSE28829 and validated the results in GSE43292 and GSE41571 separately, which could completely avoid the batch effects induced by integrated matrices and simultaneously enhance the reliability of the results.

First, we performed GSEA analysis at the level of the entire gene set, rather than identifying the DEGs between the two states of atherosclerosis, to correlate published information about biological pathways or co-expressions in biochemical experiments with the current data and thereby uncover the collective behavior of genes (8). As we expected, the gene set of GSE28829 annotated enriched immunoinflammatory KEGG pathways in advanced atherosclerosis samples. A large amount of experimental and clinical evidence demonstrates unequivocally that inflammation and immune response are integral components of the pathogenesis of atherosclerosis (2, 3, 27). Notably, the Nod (nucleotide oligomerization domain)-like receptor signaling pathway was significantly enriched in advanced atherosclerotic plaques. The Nod-like receptor pyrin domain-containing protein 3 (NLRP3) inflammasome activation contributes to the vascular inflammatory response driving atherosclerosis formation and progression (28, 29). Inhibition of NLRP3 was reported to protect against diabetes-associated atherosclerosis *via* reducing inflammation and improving vascular function (30). In addition, pathways of chemokine, cytokine, and immune cells were also reflected in advanced atherosclerosis samples. Their roles have been extensively studied, which either help in atherosclerosis advancements or vice versa, blocking the cytokines and chemokines *via* the means of broad-spectrum inhibitors, neutralizing antibodies, application of decoy receptors, or RNA interference has been proven to be effective strategies to encumber atherosclerosis progression (31–33). Likewise, GSEA has also detected the autoimmune diseases and B cell pathway enrichments. Autoimmune diseases and atherosclerosis share a number of pathogenic similarities (34), and the incidence of atherosclerosis is observed higher among patients with autoimmune diseases (35). However, knowledge about the underlying immunogenic triggers of the autoantibody response and their effects on atherosclerosis remains limited (36, 37). Interestingly, the intestinal immune network and microbiota recognition pathways were also upregulated in atherosclerosis development, which may give us a hint of the potential interactions between the gut microbial factors and the atherogenesis modulation (38).

After understanding the immunoinflammatory context of the gene set data, we proceeded to the CIBERSORTx for the dissection of immune cell heterogeneity in bulk atherosclerotic tissue. The results showed an immune landscape that the fractions of memory B cells, gamma delta T cells, M0 macrophages, and M2 macrophages were upregulated in advanced atherosclerosis samples. Inversely, the fractions of naïve B cells, CD8+ T cells, Tregs, and monocytes were downregulated in advanced atherosclerosis samples. Consistent with previous studies that tested flow cytometry and single-cell sequencing, T cells and macrophages dominate the immune cell composition of atherosclerotic plaques (39, 40). Depending on the stimuli, uncommitted macrophages (M0) could subsequently polarize toward the pro-inflammatory M1 or anti-inflammatory M2 subsets(41). Although most of the studies have concluded that M1 macrophages are enriched in progression plaques while M2 macrophages are dominant in regressing lesions (42), there are a number of studies that show the opposite—the M2 macrophages in atherosclerosis are not always protective (43, 44). Surprisingly, M2 macrophages are more susceptible to foam cell formation than M1 macrophages, and exposure to oxidized low-density lipoprotein renders M2 macrophages pro-inflammatory (45). Moreover, in human atherosclerotic plaques, M2 macrophages show a reduced capability to handle intracellular cholesterol efflux than M1 macrophages, which will promote the formation of cholesterol crystals in the atherosclerotic plaques (46). Therefore, the phenotype switch of M2 macrophages from an anti- to a pro-inflammatory profile may exert proatherogenic activities. Tregs have been shown to present within atherosclerotic plaques, and there has been a growing interest in the role of Tregs in atherosclerotic pathophysiology (47). Subsequent studies address that Tregs exert a protective role by releasing anti-inflammatory cytokines (IL-10/TGF-β) and suppressing auto reactive T cells (48, 49). Of note, co-cultured with Tregs, monocytes exhibit classical characteristics of M2 macrophages such as increased CD206 (mannose scavenger receptor) and CD163 (hemoglobin scavenger receptor, also one of the cluster genes in our study) expression. Moreover, macrophages co-incubated *in vitro* with Tregs exhibited an impaired capacity to respond to pro-inflammatory lipopolysaccharide as followed by both decreased production of IL-6 and TNF-α and decreased NF-kB activation (50). The existence of these correlations between Tregs and macrophages may explain the altered fractions of infiltrating immune cells in advanced atherosclerotic plaques in our study, favoring atherogenesis. However, their clinical confirmation in human studies remains limited. Regarding the potential use of these cells for immunomodulation, our study provided clinical evidence of the shifting roles of Tregs and macrophages in the progression of plaques, and we hypothesized that the use of exogenously cultured and functional Tregs may contribute to combatting both the defective cell capability and decreased frequency observed in atherosclerosis.

We further attempted to identify the DEGs and the PPI network underlying the progression of atherosclerosis, which listed the nine upregulated cluster genes including *ITGB2, C1QC, LY86, CTSS, C1QA, CSF1R, LAPTM5, VSIG4*, and *CD163*, most of which encode cellular membrane proteins that are instrumental to signal transduction across membranes and chemical processing of incoming molecules (51, 52). The GO analysis revealed the biological alterations in the pathogenesis of atherosclerosis. Importantly, inflammatory response and innate immune response were the most prominent terms in the BP annotation of our study, in line with the results of GSEA analysis, indicating the potential links between the cluster gene module and immunomodulatory reactions. We further performed the Pearson analysis and found that the expression of the cluster genes had a strong correlation, especially with T cells and macrophages. The classical complement C1q, mainly produced by macrophages *in vivo* (53), can promote macrophage survival during ingestion of excess cholesterol and improves foam cell efferocytosis function, which provides insight into the protective role of C1q in early atherosclerosis(54, 55). However, C1q was recently reported to be positively associated with coronary artery disease and could be considered as one of the indicators of cardiovascular outcomes (56, 57), and this was also reflected in our study that enhanced expression of C1q (*C1QC* and *C1QA*) may be a contributory factor to instability or rupture of atherosclerotic plaques. Additionally, previously published results suggested a strong association between hemoglobin-haptoglobin scavenger receptor CD163 expression in alternative macrophages and atherosclerotic disease progression, leading to intimal angiogenesis, promoted vascular permeability, and plaque inflammation (58, 59). Moreover, suppressing colony-stimulating factor-1 receptor (*CSF1R*) was reported to inhibit macrophage proliferation so as to slow down the progression of atherosclerosis (60, 61). Based on these facts, our study has the merit of providing a holistic view of the immune cells infiltration landscape correlating with gene expression and atherosclerosis severity, respectively. Most of these have not yet been reported and potential research initiatives could be raised to verify the underlying mechanisms with further studies in depth.

Atherosclerosis is a chronic inflammatory condition in which cholesterol accumulates within the subendothelial layer of the vascular wall (62). High-intensity statin treatment and lifestyle management can act to slow down the disease progression but ultimately it will not be completely halted due to its association with aging and inflammation (63). Ruptures of atherosclerotic plaques and consequent acute cardiovascular complications are still the main drivers of morbidity and mortality worldwide (64). Therefore, we have to focus on plaque composition and vulnerability rather than on plaque size and stenosis severity. In an attempt to unify the understanding of the complex paradigm of plaque destabilization and thrombogenicity in the whole process of disease progression, we enrolled two external cohorts to validate our results. GSE43292 and GSE41571 are both microarray datasets and the samples have similar histological segments in accordance with GSE28829. Although many cells are involved in the development and progression of atherosclerosis, macrophages are fundamental contributors and major immune cell populations in atherosclerotic lesions (13, 65). Based on this histological inclusion relationship, the total RNA data generated on plaque samples (GSE28829, GSE43292) has included the information about macrophages (GSE41571). Moreover, these two datasets together can be seen as the entire process of atherosclerosis from inset to rupture. Surprisingly, almost the entire cluster of genes were observed remarkably upregulated in both datasets as atherosclerosis progressed. Meanwhile, as demonstrated by the ROC curves, the AUC values of the two variables selected by LASSO regression, *C1QA* and *ITGB2*, both showed good performance in distinguishing the samples with different statuses. Hence, the cluster genes identified in this Study may be Crucial for the Development of Atherosclerosis, and Their Disease-Associated Dynamic Molecular Processes and their regulations with immune cells will hold potential insights for the investigation of curative therapies.

Naturally, there exist some inevitable limitations in this study that should be taken into consideration. For instance, the number of atherosclerosis-related datasets collected from the open public database was limited, and some of the results remain elusive because animal or human data illustrating a cause-and-effect relationship is lacking. In addition, the infiltrating immune cell subsets may display disparity when it comes to their prevalence in morphological compartments of the vessels (66). Nevertheless, the approaches and ideas in this study warrant continued investigation into the functional implications of identified genes and immune cells in human atherosclerosis. Of course, following downstream analysis of molecular and cellular experiments with strict protocols should be performed to assess their characteristics in the future.

## Data Availability Statement

The datasets presented in this study can be found in online repositories. The names of the repository/repositories and accession number(s) can be found in the article/Supplementary Material.

## Ethics Statement

Ethical review and approval was not required for this study in accordance with the local legislation and institutional requirements.

## Author Contributions

JX, CC, and YY were involved in the conception of this study and checked the manuscript. JX and CC analyzed the dataset and prepared the figures and tables. All authors have read and agreed to the published version of the manuscript.

## Funding

This work was supported by the Chinese Academy of Medical Sciences (CAMS) Innovation Fund for Medical Sciences (CIFMS, 2016-I2M-1-009).

## Conflict of Interest

The authors declare that the research was conducted in the absence of any commercial or financial relationships that could be construed as a potential conflict of interest.

## Publisher's Note

All claims expressed in this article are solely those of the authors and do not necessarily represent those of their affiliated organizations, or those of the publisher, the editors and the reviewers. Any product that may be evaluated in this article, or claim that may be made by its manufacturer, is not guaranteed or endorsed by the publisher.
